# Effects of Prebiotics and a Synthetic Microbiome Consortium on the Composition and Metabolites of the Elderly Gut Microbiota In Vitro

**DOI:** 10.1021/acs.jafc.5c00364

**Published:** 2025-05-05

**Authors:** Huimin Ye, Dara Meehan, Suzanne Timmons, Paul W. O’Toole

**Affiliations:** † School of Microbiology, and APC Microbiome Ireland, University College Cork, Western Road, T12 Y337 Cork, Ireland; ‡ Centre for Gerontology and Rehabilitation, School of Medicine, University College Cork, Western Road, T12 Y337 Cork, Ireland

**Keywords:** prebiotics, synthetic consortium, aging, colon model, gut microbiota, older person

## Abstract

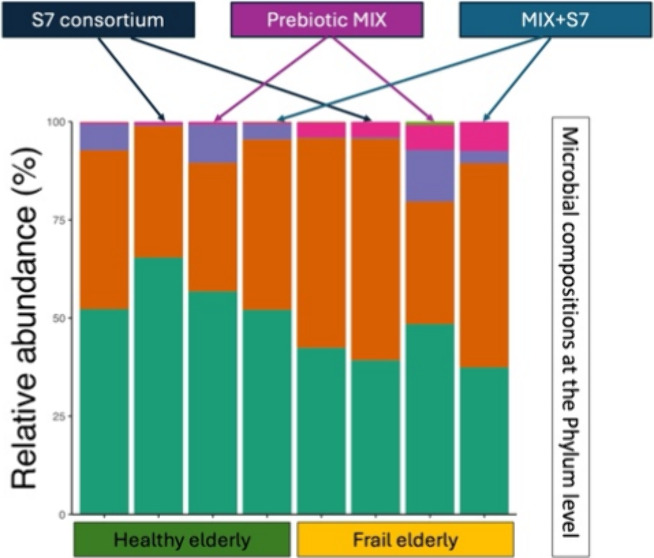

A 24 h artificial colon fermentation was performed to assess the effects of a prebiotic MIX and synthetic consortium (S7) on gut microbiota composition and microbial metabolite production in clinically stratified older adults (healthy and frail). Treatments included supplementation of the donor microbiota with a synthetic microbial consortium (S7), a prebiotic mix (MIX), and a combination of MIX and S7 (MIX+S7). The S7 treatment decreased the alpha diversity of long-stay-dwelling donor (LS, “frail”) microbiota and increased the relative abundance of S7 taxa at 16 h. MIX alone caused the enrichment of reportedly beneficial genera such as *Coprobacillus* and *Eubacterium* in community-dwelling donor (CM, “healthy”) microbiota and *Citrobacter* and *Faecalibacterium* in LS microbiota. The MIX+S7 treatment sustained higher overall S7 species richness and consortium taxon abundance at 24 h. Both MIX and MIX+S7 treatments enhanced short-chain acid production compared to control. These findings highlight the differential responses of microbiota from distinct elderly health strata to prebiotic and microbial consortia supplementation.

## Introduction

The world is experiencing an unprecedented demographic shift: populations are aging at an accelerating pace. By 2050, the number of individuals aged 60 and older is expected to double, reaching over 2 billion globally.^1^ A main focus on global aging is achieving “healthy aging”, which is the process of developing and maintaining the functional ability that enables well-being in older age.^2^ One of the most significant obstacles to healthy aging is frailty, a clinical condition characterized by decreased physiological reserve and increased vulnerability to stressors.^3^

Aging is associated with significant physiological changes, including alterations in the composition and function of the gut microbiota.^4,5^ These changes often involve reducing microbial diversity and the depletion of health-associated taxa, linked to impaired gut barrier function, chronic low-grade inflammation, and an increased risk of age-related diseases.^6−9^ Research has highlighted distinct microbiota profiles in community-dwelling (CM, “healthy”) and long-stay-dwelling (LS, “frail”) older adults.^10^ The LS population often exhibits greater dysbiosis, characterized by reduced microbial diversity, loss of health-associated commensal species, and the out-growth of potentially harmful taxa, sometimes called pathobionts.^9,11^

Given the central role of the gut microbiota in modulating host health through effects on innate immunity, metabolism, and the brain-gut axis,^5,12^ interventions aimed at restoring or maintaining a beneficial microbial community have garnered growing interest as strategies to promote healthy aging. Among potential interventions, prebiotics—nondigestible dietary compounds that selectively stimulate the growth of beneficial bacteria^13^ —and synthetic microbial consortia composed of defined strains with known functional properties^14^ are particularly promising. These approaches have been shown to enhance the abundance of health-associated microbial taxa, increase the production of beneficial metabolites such as short-chain fatty acids (SCFAs), and improve gut barrier integrity.^15−18^ Despite their potential, the efficacy of prebiotics and microbial consortia can vary depending on the baseline gut microbiota composition of individuals, which is influenced by health status, diet, and living conditions.^19,20^ CM and LS subjects exhibit distinct microbiota profiles, with the latter showing signs of greater dysbiosis.^6^ This variation underscores the importance of tailoring interventions to specific populations to maximize therapeutic benefits.

To better understand the potential of these interventions, this study investigated the effects of prebiotics and a synthetic microbial consortium (S7) for healthy aging developed in our laboratory^21^ on the gut microbiota of older adults using an in vitro artificial colon fermentation model. Stools from CM and LS older adults were used to inoculate bioreactors, simulating the gut environment under controlled conditions. Treatments included basal medium supplemented with S7 (S7), a prebiotic mix (MIX), or a combination of MIX and S7 (MIX+S7). The aim was to evaluate the impact of these treatments on microbiota diversity, community structure, and metabolite production, as well as to explore how differences in donor microbiota composition influence treatment outcomes. By comparing the responses of CM and LS microbiota, this study provides new insights into the potential for prebiotics and synthetic microbial consortia to modulate the gut microbiota of older adults, paving the way for targeted strategies to promote healthy aging.

## Materials and Methods

### Fecal Sample Collection and Preparation of Fermenter Inocula

This study was approved by the Clinical Research Ethics Committee of the Cork Teaching Hospitals (study number APC134), and informed consent was obtained from all participants. Exclusion criteria comprised the presence of a significant coexisting malignant disease or end-stage organ disease, participation in an investigational drug study, antibiotic treatment within the last 30 days, ongoing warfarin therapy, continuous use of nonsteroidal analgesics, regular administration of oral or intravenous corticosteroids, major intestinal surgery within the past two years, presence of an ileostomy or colostomy, diagnosis of inflammatory bowel disease or irritable bowel syndrome, and current or historical alcohol abuse. No use of prebiotic or probiotic supplements was reported by any of the subjects prior to sampling, based on dietary assessment using a food frequency questionnaire. Two eligible donors from each group—community-dwelling (CM2 female 72 years old (years), CM3 male 78 years) and long-stay residential care (LS6 female 75 years, LS7 female 89 years)—were selected based on their microbiota composition by mapping their beta diversity with that of ELDERMET subjects in our previous study.^6^ The Rockwood Clinical Frailty Scale rating was used to determine their degree of frailty.^22^ Fresh fecal samples were collected and transported to the lab within 1 h after defecation. Upon arrival, the samples were immediately transferred into an anaerobic chamber (Don Whitley Scientific, UK). The fecal material was then homogenized and diluted (1:10 w/v) with a 20% sterile glycerol/PBS solution before being stored at −80 °C.

### Preparation of the S7 Consortium Inoculum

A previously identified healthy-aging-related synthetic consortium (“S7”) was used for the in vitro fermentation.^21^ The S7 consortium consists of *Alistipes putredinis*, *Barnesiella intestinihominis*, *Coprococcus catus*, *Dorea longicatena*, *Agathobacter rectalis* (previously*Eubacterium rectale*),^23^*Faecalibacterium prausnitzii*, and *Roseburia hominis*. The cultures of the S7 taxa were grown from frozen stocks in a modified YCFA medium^24^ separately to reach their maximum OD values. Each volume of S7 species culture was mixed at the same OD values (OD_600_ = 0.5) and centrifuged at 5000 rpm at 4 °C for 10 min. The pellet was resuspended with 1/2 volume of reduced sterile PBS and glycerol (20% v/v) and stored at −80 °C until use. All the work was performed in the anaerobic chamber except the centrifugation step.

### In Vitro Fermentations

Conditions mimicking the human colon were recreated in a single-stage continuous fermentation system (MiniBio Reactors, Applikon Biotechnology), as previously described.^24^ One percent (w/v) fecal inoculum was used to inoculate four parallel single fermentation vessels with a 150 mL working volume of basal medium.^25^ The impacts of a prebiotic MIX (Supplementary Table 1), the S7 consortium, and their combination (MIX+S7) on the gut microbiota of older individuals were investigated through supplementation. The system was set to a temperature of 37 °C, pH was regulated at 6.8, and stirring was maintained at 100 rpm. Anaerobic conditions were maintained through the delivery of N_2_ gas over a period of 24 h following protocols used in previous studies of prebiotic effects on fecal microbiota.^25,26^ At 0, 16, and 24 h, 5 mL of fecal slurry samples were collected and centrifuged to separate the supernatant and pellet. Pellets and supernatants were stored separately at −20 °C for further DNA extraction and short-chain fatty acids (SCFAs) analysis, respectively.

### DNA Extraction of Fecal Pellets and Fermenter Samples

To profile the gut microbiota in donor samples or the fermentation medium, the total DNA of fecal pellets or fermenter samples was isolated for 16S rRNA gene amplicon sequencing. Fecal pellets were thawed on ice and resuspended in the residual liquid. A 250 μL aliquot of the resuspended pellet was used for DNA extraction. DNA was extracted using a QIamp Fast DNA Stool kit (Qiagen) with modifications. The samples were processed by bead-beating with 600 μL of preheated InhibitEX Buffer, using alternating cycles of 1 min bead-beating and 1 min cooling on ice, followed by a final 30 s bead-beating step. The mixture was then incubated on a heat block at 70 °C for 10 min. After brief vortexing and centrifugation at full speed, 250 μL of the supernatant was transferred to a sterile Eppendorf tube. Proteinase K (15 μL) and 200 μL of AL buffer were added, and the mixture was incubated at 70 °C for 10 min. After 200 μL of ethanol was added, the lysate was transferred to a QIAmp Spin column. The column was centrifuged for 1 min, washed with 500 μL of Buffer AW1, and centrifuged again. A second wash was performed with 500 μL of Buffer AW2, followed by final centrifugation for 3 min to remove any remaining liquid. The DNA was eluted from the column in 70 μL of AE buffer, incubated at room temperature for 1 min, and centrifuged for 1 min.

### 16S rRNA Gene Amplicon Sequencing

A ZymoBIOMICS Microbial Community Standard II (log distribution) (Zymo Research, Tustin, CA, USA) was included as the positive control. The library preparation and amplicon sequencing were conducted by Novogene (Cambridge, UK). Briefly, primers (CCTAYGGGRBGCASCAG, GGACTACNNGGGTATCTAAT) targeting the V3–V4 region of 16S rRNA were used for PCR amplification, along with barcodes. The PCR products were validated by 2% agarose gel electrophoresis. An equal amount of PCR products from each sample were pooled, subjected to end-repaired and A-tailing, and ligated with Illumina adaptors. The resulting libraries were sequenced using a paired-end Illumina platform to produce 250bp paired-end raw reads. Library quality was then evaluated and quantified via qPCR. The quantified libraries were pooled and sequenced on an Illumina PE250 platform, yielding between 53,603 and 136,423 raw reads per sample. Paired-end reads were assigned to each sample based on the unique barcodes, after which the barcode and primer sequences were removed. Pair-end reads were merged using FLASH^27^ and chimera sequences were removed using UCHIME algorithm.^28^

### Microbiota Composition Analysis

16S rRNA gene amplicons were processed using the DADA2 package (version 1.14.0),^29^ and produced amplicon sequence variants (ASVs) were classified using SILVA 138.1 database.^30^ Sequencing data were analyzed using the software packages *Phyloseq* (version 1.48.0)^31^ and *microeco* (version 1.7.1)^32^ in R (version 4.4.0). For alpha- and beta-diversity analyses, the ASV tables were rarefied at the depth of 42,207 read counts. Differential analysis was performed using DESeq2.^33^

### Short-Chain Fatty Acids Measurement

The supernatant from the fermentation samples was thawed and transferred into Spin-X centrifuge tubes with a 0.22 μm filter and centrifuged for 10 min at 6000*g* at 4 °C. The resulting filtrate was then used to measure short-chain fatty acids (SCFAs). SCFAs were quantified using an Agilent 1200 HPLC system (Agilent Technologies, CA, USA) with a REZEX 8% H+ organic acid column (300 × 7.8 mm, Phenomenex, CA, USA) maintained at 65 °C. The mobile phase consisted of 0.01 N H_2_SO_4_, with a 0.6 mL/min flow rate. Organic compounds were detected using a refractive index detector. Reference standards of SCFAs and organic acids, including glucose, lactose, lactic acid, formic acid, succinic acid, propionic acid, acetic acid, and butyric acid, each at a concentration of 10 mM, were used for data analysis.

### Statistical Analysis

ANOVA with post hoc Duncan’s multiple range test was performed to identify significant alpha diversity changes. Beta diversity was compared among treatments using permutational multivariate analysis of variance based on the adonis2 function of vegan package.^34^ The DESeq2 package was used to identify differential taxa between the treatment of prebiotics MIX and basal control at the genus level. The *p-*value for DESeq2 results was adjusted for multiple comparisons using the Benjamini–Hochberg method, and results were considered significant if the adjusted *p*_adj_ < 0.05. One-way ANOVA and Holm–Sidak’s multiple comparison tests were applied using GraphPad Prism version 10 (GraphPad) to compare SCFAs production between the fecal microbiota after 16 and 24 h fermentation with prebiotic MIX and MIX+S7 supplements.

## Results

**Figure 1:**
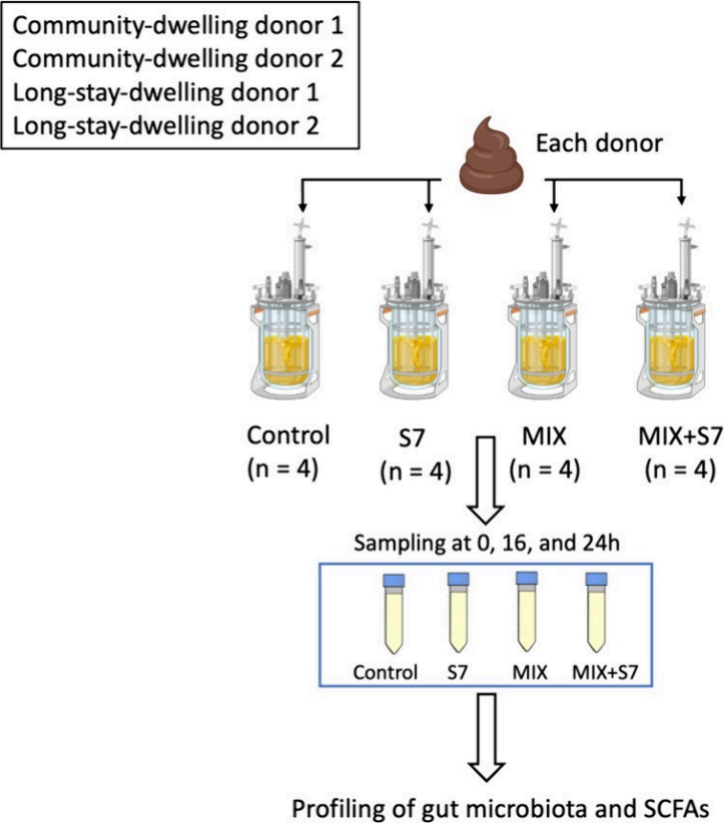
Experimental design of the in vitro fermentation experiments. Stools from two community-dwelling (CM) and two long-stay-dwelling (LS) older adult were used as independent inocula. For each treatment condition—(1) basal medium (control), (2) basal medium + S7 consortium, (3) basal medium + prebiotics MIX, (4) basal medium + S7 consortium + prebiotics MIX—four replicate bioreactors were inoculated per donor. This resulted in 8 bioreactors (2 donors × 4 replicates) for CM and LS subjects per treatment. The fermentation experiments were conducted for 24, which sampling at 0, 16, and 24 h for subsequent microbial and metabolic analyses.

In this study, we evaluated the potential of a prebiotic MIX and/or a synthetic seven-species consortium (S7) linked to healthy aging to modulate microbiota composition and functional outputs, such as short-chain fatty acids (SCFAs) production, during in vitro fermentation (see Figure 1 for experimental design). The investigation aimed to determine whether these interventions could support beneficial microbiota shifts relevant to healthy aging.

### Validation of Gut Microbiota Representativeness of Frail and Healthy Fecal Donors

Four donors were selected for the fermentation: two community-dwelling (CM) donors representing ‘healthy’ microbiota and two long-stay-dwelling (LS) donors representing ‘frail’ microbiota. Comparison of their fecal microbiota with that of previously characterized ELDERMET cohort CM and LS subjects revealed different microbial compositions related to frailty (Supplementary Figure 1). At the phylum level, the LS donors exhibited a lower relative abundance of Firmicutes and Bacteroidota, and a higher relative abundance of Actinobacteria and Proteobacteria compared to CM donors (Supplementary Figure 2a). At the family level, *Bifidobacteriaceae*, *Enterobacteriaceae*, *Rikenellaceae*, *Lactobacillaceae*, and *Coriobacteriaceae* were more abundant in LS subjects relative to CM subjects (Supplementary Figure 2b). At the genus level, *Bifidobacterium*, *Escherichia-Shigella*, and *Ligilactobacillus* were enriched in LS donors, while *Bacteroides*, *Blautia*, *Faecalibacterium*, *Ruminococcus*, and *Agathobacter* were more abundant in CM donors (Supplementary Figure 2c). These findings are consistent with our previous results, which identified *Faecalibacterium* and *Agathobacter* as key taxa enriched in CM donors.^6^ Similarly, in line with our study,^6^ the microbiota alpha diversity, measured by the Shannon index, was significantly lower in LS donors than in CM donors (Supplementary Figure 2d).

At baseline, the microbiota clustered by donor regardless of treatments, with two CM donors clustering closely together, while the LS donors’ microbiota displayed greater divergence (Supplementary Figure 2e).

### Prebiotic MIX and S7 Consortium Impacted the Alpha and Beta Diversity of CM and LS Microbiota

**Figure 2:**
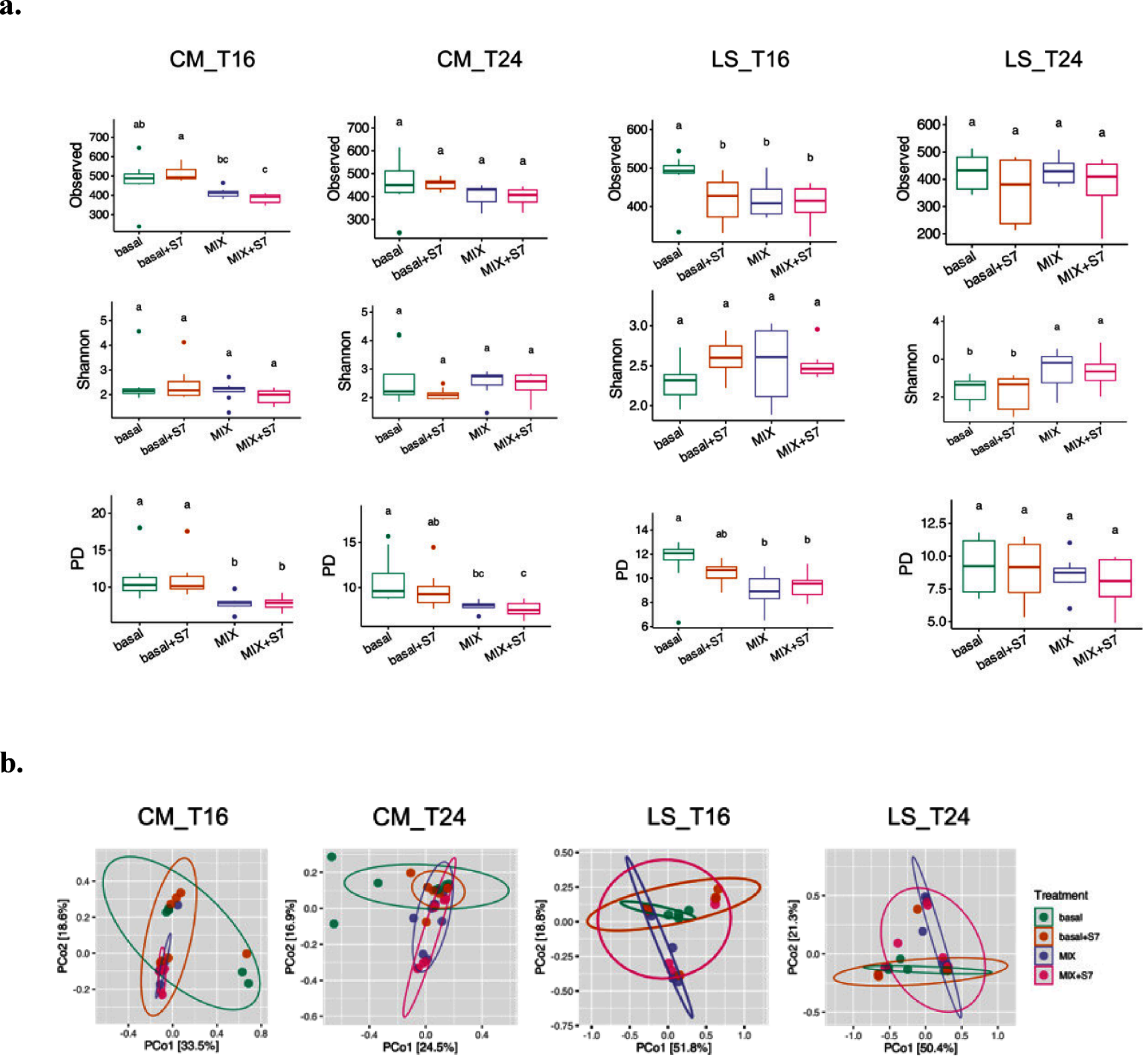
Alpha and beta diversity at T16 and T24. (a) Box plots showing alpha diversity metrics—observed species, Shannon, and phylogenetic diversity (PD) index—for each group at 16 h (T16) and 24 h (T24). Different letters (a, b, c) indicate statistically significant (*P* < 0.05) differences between groups. (b) Beta diversity at T16 and T24. Principal coordinates analysis (PCoA) plots based on Bray–Curtis distances. Each dot represents a sample collected after 16 h (T16) or 24 h (T24) of fermentation. Color coding for experimental conditions: green (basal medium), orange (basal + S7), purple (prebiotic MIX), and magenta (prebiotic MIX + S7). CM, community-dwelling; LS, long-stay-dwelling.

Supplementation of the fermentation with MIX+S7 significantly reduced the number of Observed Species in CM microbiota at T16 (Figure 2a). All three treatments (basal+S7, MIX, and MIX+S7) lowered the number of observed species in LS at T16. The effects of those treatments on Observed Species numbers were not maintained at T24 in the fermentations of either CM or LS donors. The treatment with either MIX or MIX+S7 significantly increased the Shannon index of LS microbiota at T24. A reduced level of phylogenetic diversity (PD) was observed in both CM and LS seeded fermentations supplemented with MIX and MIX+S7 at T16 and this effect remained in CM microbiota at T24. At the level of the overall microbiome composition, beta diversity analysis based on Bray–Curtis distances revealed no effect of S7 supplementation when comparing basal+S7 to basal and MIX+S7 to MIX (Figure 2b and Supplementary Table 2). Treatment with the prebiotic MIX significantly altered the CM microbiota composition measured by beta diversity compared with basal control at T16 and T24 (Figure 2b and Supplementary Table 2).

**Figure 3:**
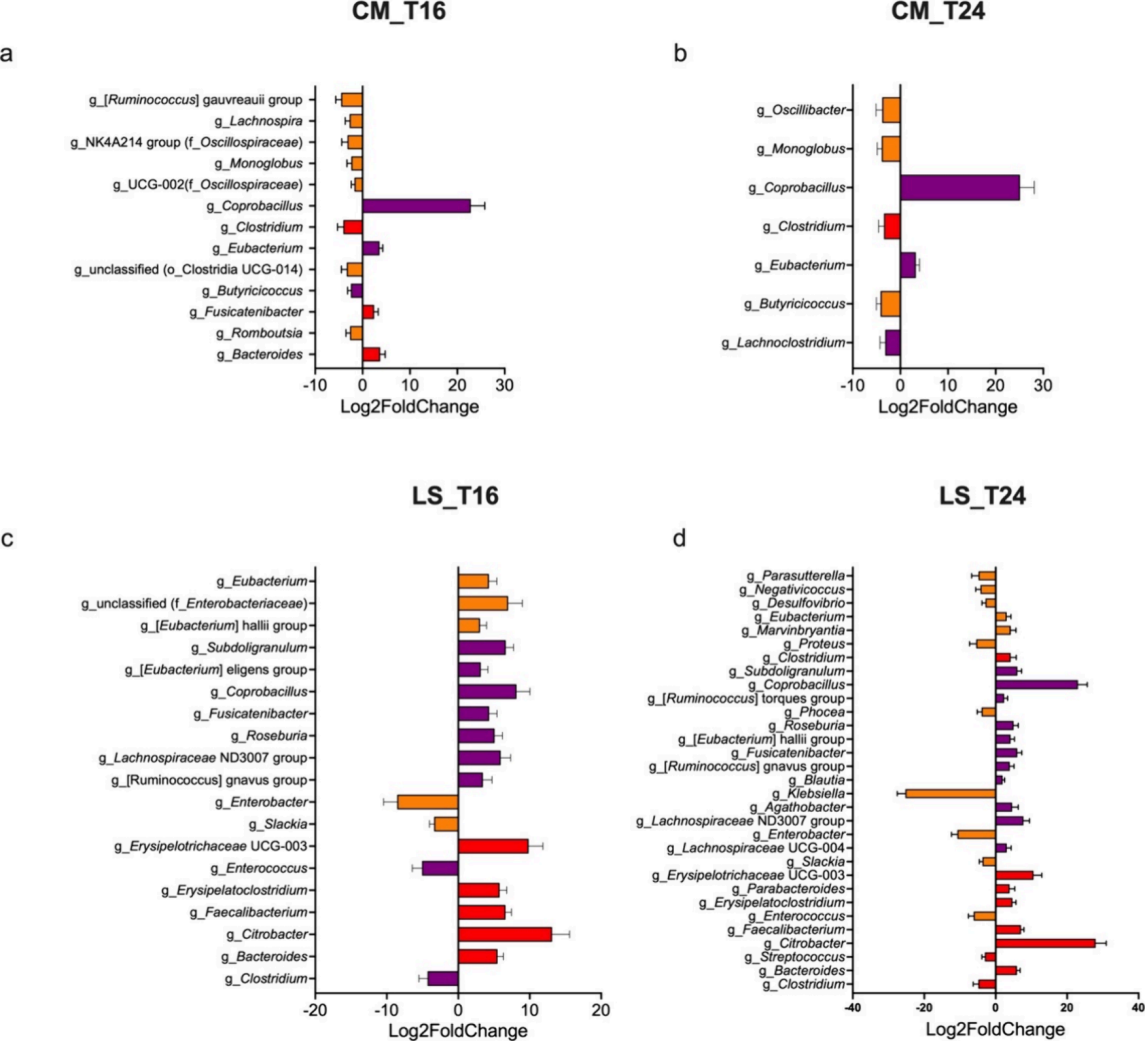
Differentially abundant genera between MIX and basal control. Differential taxa in the gut microbiota of CM donors at 16 and 24 h (a and b). Differential taxa in the gut microbiota of LS donors at 16h and 24h (c and d). Differentially abundant taxa were identified using DESeq2 with an adjusted *p*-value of <0.05. Genera to the right and the left of the zero line are more and less abundant in MIX treatment than basal controls, respectively. Bacterial genera were classified as dominant (red, relative abundance ≥1%), low abundance (purple, relative abundance between 0.1 and 1%), or rare (orange, relative abundance ≤0.1%) based on their relative abundance in MIX treatment group. CM, community-dwelling; LS, long-stay-dwelling.

To determine the compositional differences underlying these alterations, we identified differentially abundant genera between MIX and basal controls using the program DESeq2. Supplementation with the prebiotic MIX significantly increased the relative abundances of *Coprobacillus* and *Eubacterium* while reducing the relative abundances of *Clostridium*, *Monoglobus*, and *Butyricicoccus* in CM microbiota at T16 and T24 (Figure 3a,b). The relative abundances of dominant genera (relative abundance >1%), including *Bacteroides*, *Citrobacter*, *Faecalibacterium*, *Erysipelatoclostridium*, and *Erysipelotrichaceae* UCG-003, were increased by supplementation with the prebiotic MIX in LS microbiota (Figure 3c,d). Prebiotic MIX treatment consistently reduced the abundances of *Enterobacter*, *Slackia*, *Enterococcus*, and *Clostridium* in LS microbiota at 16 and 24 h.

### Supplementation with the S7 Consortium Increased the Richness and Relative Abundance of S7 Species

**Figure 4:**
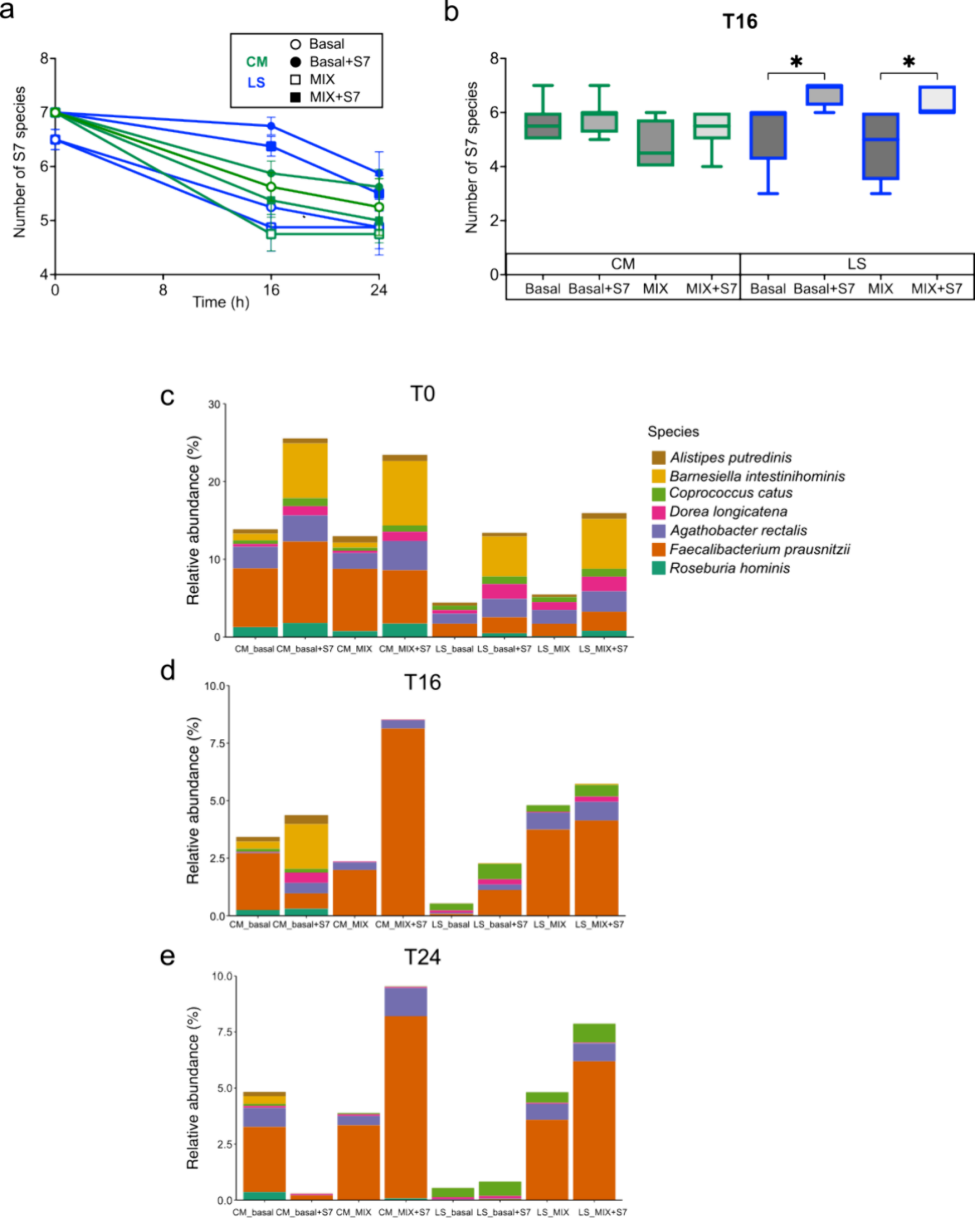
Richness and abundances of S7 species in experimental fermentations. (a) The number of S7 species present over time was evaluated by BLAST analysis of amplicon sequence data with the 16S rRNA sequences of S7 species. (b)Number of S7 species detected in each treatment group at T16 using BLAST. Significant differences in S7 species number were identified using ANOVA with Holm–Sidak’s multiple comparisons test. * *P* < 0.05. Averaged relative abundance of S7 species in different treatment groups at T0 (c), T16 (d), and T24 (e). CM, community-dwelling; LS, long-stay-dwelling.

The number of S7 species detected decreased over fermentation time when not supplemented, but not surprisingly both basal+S7 and MIX+S7 showed higher numbers of S7 species compared to basal and MIX at T16 and T24 (Figure 4a). The basal+S7 and MIX+S7 treatments significantly increased the number of S7 species in LS donor microbiota compared to basal and MIX at T16, respectively (Figure 4b). The combined relative abundance of S7 species was lower in LS donor microbiota compared to CM donor microbiota (Figure 4c, LS_basal vs CM_basal) at T0. Supplementation of S7 in the CM donor not only increased the relative abundance of the S7 consortium but also maintained the S7 consortium richness at T16 (Figure 4d, CM_basal+S7 vs CM_basal). The treatment with S7 of LS donor microbiota also enhanced the richness of the S7 consortium and its combined relative abundance at T16, particularly for *Coprococcus catus*, *Dorea longicatena*, *Agathobacter rectalis*, and *Faecalibacterium prausnitzii*. The effects of S7 supplementation on both CM and LS microbiota were not sustained at T24.

Supplementation with the prebiotic MIX maintained the richness of the S7 consortium and increased the consortium relative abundance in LS microbiota compared to basal control at T16 and T24 (Figure 4d,e). In contrast, prebiotic MIX reduced the richness and combined relative abundance of the S7 consortium in CM microbiota at T16 and T24. The combination of prebiotics MIX and the S7 consortium increased the combined relative abundance of S7 in CM microbiota while reducing its richness by favoring the growth of *F. prausnitzii* and *A. rectalis* (Figure 4d,e). MIX+S7 treatment in CM microbiota increased the total relative abundance of the S7 consortium while reducing the richness. The enhancement of S7 richness and combined relative abundance was observed in LS microbiota treated with MIX+S7 (Figure 4d,e).

### Effect of Prebiotic MIX and S7 Consortium on SCFAs Production in the Fecal Microbiota Fermentations of Elderly Subjects

**Figure 5:**
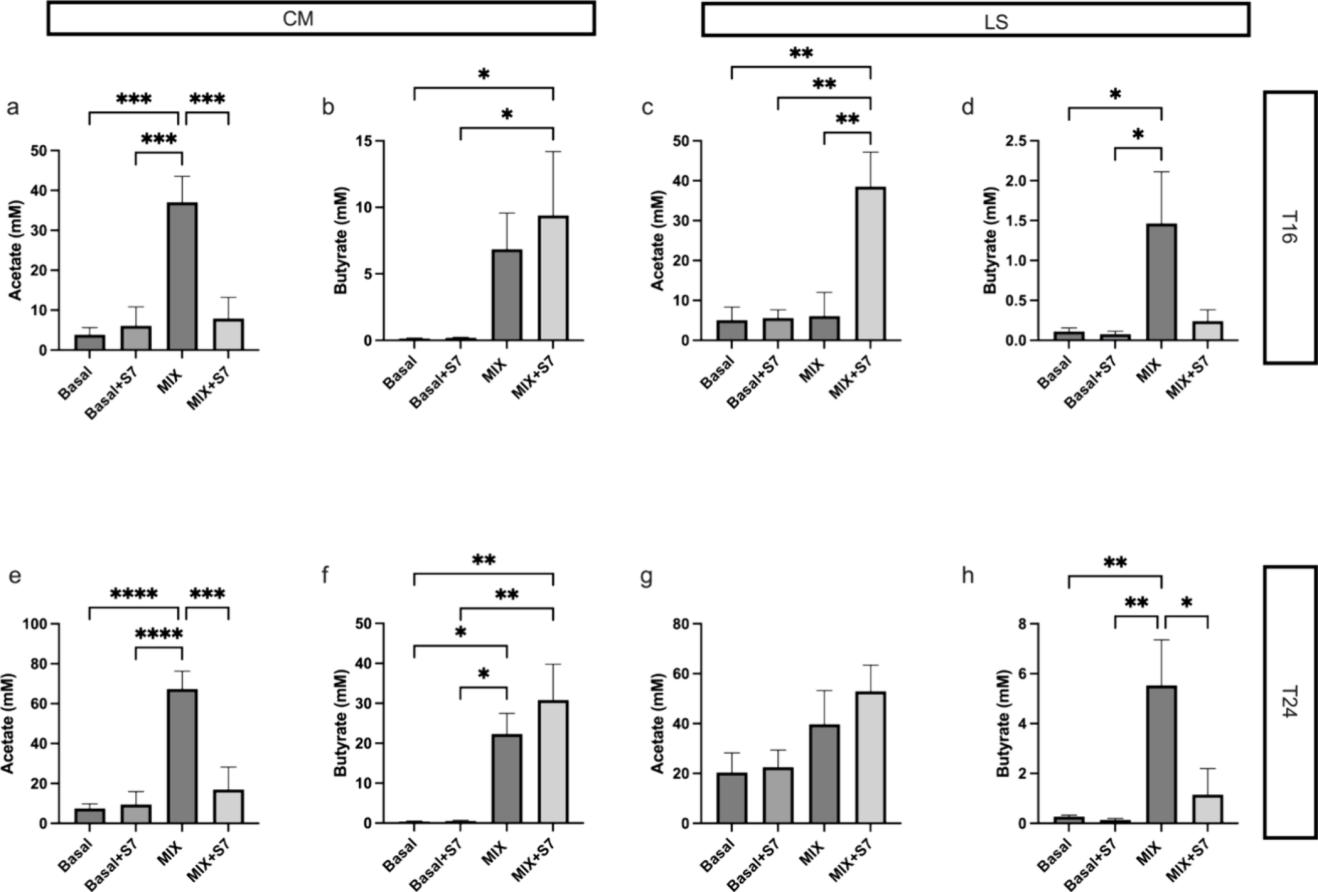
(a–h) Concentrations of short-chain fatty acids (SCFAs) in the supernatant after 16 h (T16) and 24 h (T24) fermentation. Acetate and butyrate concentrations were compared between treatments using one-way ANOVA and Holm-Sidak’s multiple comparison test. * *P* < 0.05, ** *P* < 0.01, *** *P* < 0.0001, **** *P* < 0.00001. Bars are plotted with mean ± SEM. CM, community-dwelling; LS, long-stay-dwelling.

Supplementation with the S7 consortium alone showed no effect on acetate and butyrate production in CM or LS microbiota (Figure 5a–h). The Prebiotic MIX significantly increased the acetate concentration produced by CM microbiota compared to basal control at T16 and T24 (Figure 5a,e). Additionally, prebiotic MIX supplementation significantly enhanced butyrate production by CM microbiota at T24 (Figure 5f). The treatment with prebiotic MIX did not impact the acetate concentrations produced by LS microbiota (Figure 5c,g). However, prebiotic MIX supplementation of LS microbiota significantly increased the concentration of butyrate (Figure 5d,h). Treatment with MIX+S7 increased butyrate production in CM microbiota at T16 and T24 (Figure 5b,c) while significantly enhancing acetate production in LS microbiota at T16 (Figure 5c). To investigate the effects of the S7 consortium in the presence of the prebiotic MIX, we compared the MIX+S7 treatment to MIX alone. In the CM microbiota, MIX+S7 significantly reduced acetate production compared to MIX. In the LS microbiota, supplementation with MIX+S7 led to a significant increase in acetate production at T16 and a reduction in butyrate production at T24 relative to MIX. These findings underscore the contrasting effects of the S7 consortium on SCFAs production in CM and LS microbiota when combined with the prebiotic MIX.

### Microbial Taxa Responsive to Prebiotic MIX and S7 Consortium

**Figure 6:**
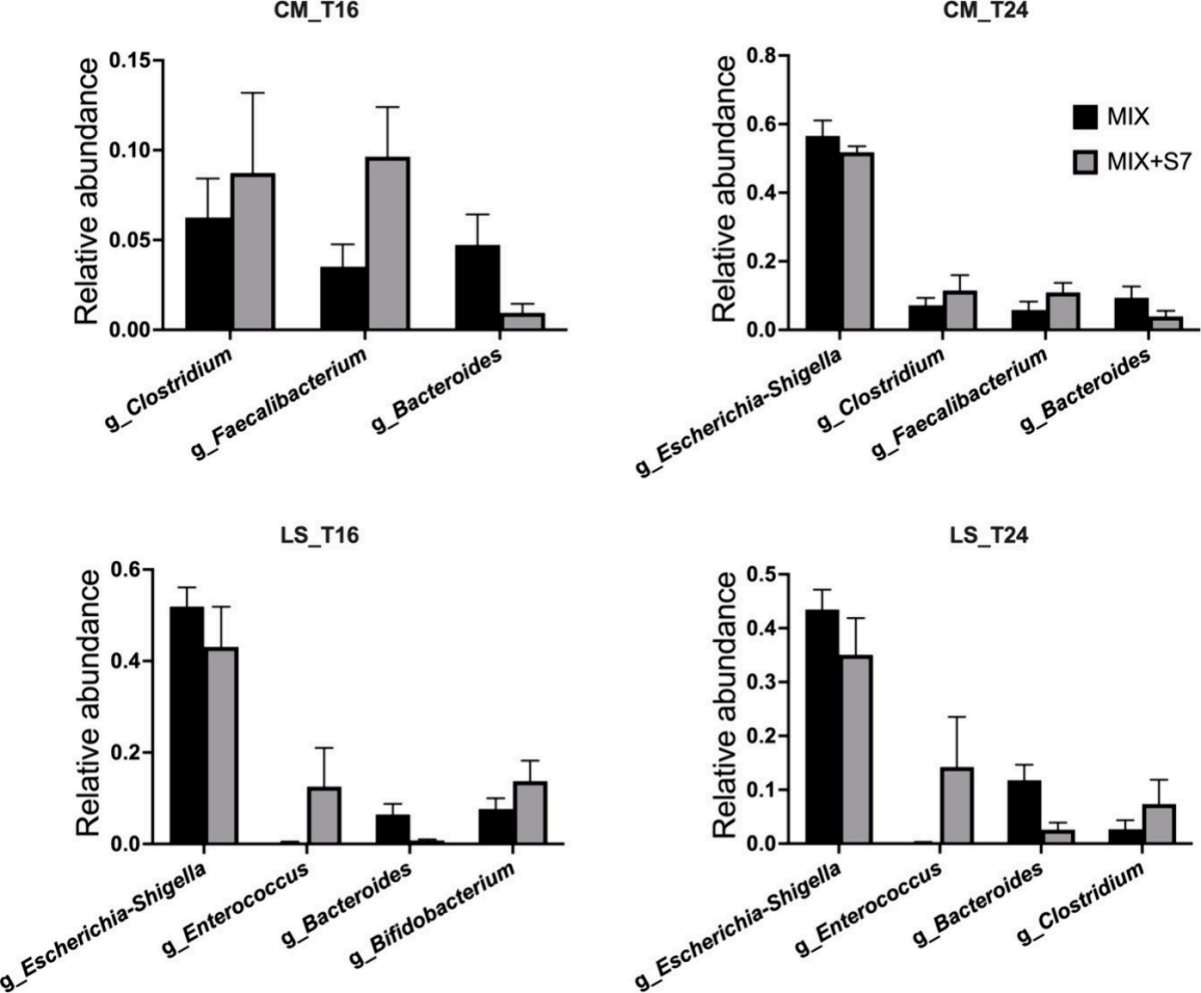
Differential taxa between MIX+S7 and MIX at T16 and T24. Differential taxa between MIX and MIX+S7 were identified using unpaired *t*-tests with the Bonferroni–Dunn method for *p*-value correction. Only significantly different taxa are shown (*P* < 0.05). Bars are plotted with mean ± SEM. CM, community-dwelling; LS, long-stay-dwelling.

To identify the differentially abundant taxa between MIX and MIX+S7, we performed a *t*-test on the relative abundances at the genus level. Supplementation with MIX+S7 of the LS microbiota significantly reduced the relative abundances of *Escherichia-Shigella* and *Bacteroides* and increased the relative abundances of *Enterococcus* and *Bifidobacterium* at T16 and T24 (Figure 6). Treatment with MIX+S7 of the CM microbiota fermentation consistently increased the relative abundance of *Clostridium* and *Faecalibacterium* and reduced the relative abundance of *Bacteroides* at T16 and T24. Additionally, MIX+S7 in CM microbiota significantly reduced the relative abundance of *Escherichia-Shigella* at T24.

## Discussion

The present study aimed to evaluate the effects of a prebiotic MIX and a synthetic seven-species consortium (S7) on the gut microbiota of community-dwelling (CM, “healthy”) or long-stay-dwelling (LS, “frail”) older adults using an in vitro fermentation model. The findings provide valuable insights into the potential of these interventions to modulate gut microbiota for healthy aging.

The prebiotic MIX demonstrated significant modulation of the gut microbiota, with notable effects on alpha and beta diversity metrics. Specifically, supplementation with MIX increased the Shannon diversity of LS microbiota at 24 h, indicating a potential for prebiotics to enhance microbial diversity in microbiota associated with frailty. Differential abundance analysis revealed that MIX enriched genera such as *Clostridium*, *Coprobacillus*, and *Eubacterium* in CM microbiota, and *Citrobacter*, *Faecalibacterium*, and *Erysipelotrichaceae* UCG-003 in LS microbiota. Many of these taxa, such as *Clostridium*, *Eubacterium*, and *Faecalibacterium,* are associated with beneficial metabolic functions, including butyrate production, which supports gut barrier integrity and anti-inflammatory effects.^35−37^ Supplementation with MIX mainly favored the growth of *Faecalibacterium prausnitzii* and *Agathobacter rectalis* in both CM and LS microbiota compared to controls.

The S7 consortium supplementation not surprisingly increased the total relative abundance of S7 species in both CM and LS microbiota compared to controls at 16 h. A significant increase in S7 species richness with S7 treatment was observed in the LS microbiota at 16 h. This may reflect the lower ecological resilience of LS microbiota, consistent with previous findings of reduced diversity and functionality in microbiota associated with frailty.^38,39^ However, the effects of S7 treatment alone diminished by 24 h suggested that multiple doses may be required in future studies to sustain its impact. Furthermore, the limited effects of S7 supplementation on the microbiota diversity and microbial SCFAs production suggested that its primary contribution may lie in its ability to maintain or enhance the relative abundance of its constituent beneficial taxa, such as *Agathobacter rectalis*, *Coprococcus catus*, and *Faecalibacterium prausnitzii*. Notably, *Alistipes putredinis* and *Barnesiella intestinihominis* were present only in CM microbiota without treatments at 24 h, suggesting that these taxa may be naturally enriched or more abundant in the baseline, untreated state of CM microbiota. This could imply that the presence of specific microbial species is influenced by the initial composition of the microbiota and may not be as responsive to treatment conditions compared to other species in the community

The combination of MIX and S7 (MIX+S7) showed synergistic effects on microbiota composition and SCFAs production. The highest relative abundance of combined S7 taxa was observed with MIX+S7 in both CM and LS microbiota. In LS microbiota, MIX+S7 reduced the relative abundances of *Escherichia-Shigella* and *Bacteroides* while increasing *Enterococcus* and *Bifidobacterium*, compared to MIX alone. Future studies employing species- or strain-resolution methods (e.g., shotgun metagenomics or targeted culturing) are needed to resolve the precise role of *Enterococcus* in the context of MIX treatment and its potential interactions with SCFAs production. These shifts underscore the potential of the MIX+S7 combination to restore a healthier microbiota balance in frail microbiota. MIX+S7 also modulated SCFAs production differently in CM and LS subjects. For example, while acetate production was enhanced in LS microbiota, butyrate production was increased in CM microbiota at 16 h. These contrasting effects suggest that the interplay between MIX and S7 may alter the community constellation of metabolic pathways, potentially reflecting competition between microbial taxa or substrate utilization differences. Further studies are needed to elucidate the mechanisms underlying these effects.

The ability of prebiotic MIX and S7 to modulate gut microbiota composition and functional outputs has significant implications for healthy aging. Prebiotics like MIX, which promote beneficial microbial taxa and SCFAs production, could support gut and systemic health by mitigating inflammation and enhancing gut barrier integrity. The enrichment of butyrate-producing bacteria and the modulation of gut taxa by MIX+S7 further suggest its potential as a therapeutic strategy for frail individuals. However, the donor-specific responses observed in this study highlight the importance of personalized approaches in microbiota-targeted interventions. Factors such as baseline microbiota composition, ecological resilience, and substrate availability likely influence the outcomes of prebiotic and probiotic supplementation. Future studies should explore these factors in greater depth to optimize the efficacy of interventions like MIX and S7. We employed PICRUSt2^40^ to investigate potential alterations underlying the observed SCFAs changes. However, PICRUSt2 predictions were uninformative (data not shown), highlighting the need for metagenomic sequencing to unequivocally investigate functional differences in fermenter states.

This study provides valuable insights into how the S7 consortium and prebiotics modulate the gut microbiota of older donors, including both healthy and frail profiles. While MIX enhanced microbial diversity and enriched beneficial taxa, S7 maintained its constituent species and further modulated the microbiota when combined with MIX. These findings support the development of targeted microbiota interventions to promote healthy aging, with potential applications in healthy and frail older populations. However, in vitro fermentation models lack the complexity of in vivo systems, such as host-microbiota interactions and immune responses. Additionally, the short duration of fermentation limits the ability to assess long-term effects. Although we preserved interindividual variation by using independent donors (*n* = 2 per group) without pooling, the small cohort size limits the generalizability of our results to broader populations. Future validation with in vivo trials—with larger, clinically stratified cohorts—will be essential to confirm the translational potential of these interventions for healthy aging.

## Supporting Information Available

Table S1: ingredients of basal medium and prebiotic MIX; Table S2: pairwise comparison of beta diversity between treatments in CM and LS subjects at T16 and T24; Figure S1: nonmetric multidimensional scaling (NMDS) ordination of fecal microbiota in community-dwelling (CM) and long-stay-dwelling (LS) older subjects; Figure S2: microbiota compositions of donors at baseline; and Figure S3: microbial compositions after 16 and 24 h fermentation (PDF)
