# Supplementary material for: Effects of Prebiotics and a Synthetic Microbiome Consortium on the Composition and Metabolites of the Elderly Gut Microbiota In Vitro
Source: J Agric Food Chem. 2025 May 5;73(19):11720–9. doi: 10.1021/acs.jafc.5c00364 (PMC12082690; doi:10.1021/acs.jafc.5c00364)
Supplement: Supplementary file 1 — jf5c00364_si_001.pdf [file jf5c00364_si_001.pdf]

Supporting Information for

Effects of prebiotics and a synthetic microbiome consortium on the composition and metabolites of the elderly gut microbiota *in vitro*

Huimin Ye[1], Dara Meehan[1], Suzanne Timmons[2], Paul W. O'Toole[1*]

1 *School of Microbiology, and APC Microbiome Ireland, University College Cork, Western Road, T12 Y337, Cork, Ireland*

2 *Centre for Gerontology and Rehabilitation, School of Medicine, University College Cork, Western Road, T12 Y337, Cork, Ireland*

*Correspondence: Paul W. O'Toole, School of Microbiology & APC Microbiome Ireland, Food Science Building, University College Cork, T12 Y337, Cork, Ireland; Tel: +353 21 490 3997; email: pwotoole@ucc.ie

Table S1. Ingredients of basal medium and prebiotic MIX

	**Ingrdients**
Basal medium	Casein (Bact-Casitone), 3 g/l; Peptone water, 2 g/l; Yeast extract, 2 g/l; Bile salts, 0.5 g/l; Tween 80, 2ml/l; Vitamin K, 10 ul/l; 0.1% w/v Haemin solution, 10ml/l; NaHCO3, 2 g/l; Solution 1 (KH2PO4, 0.04g/l), 20 ml/l, Solution 2 (K2HPO4, 0.04g/l; NaCl, 0.1 g/l; CaCl2.6H2O; 0.01 g/l; MgSO4.7H2O, 0.01 g/l), 20 ml/l; Resazurine solution (1 mg/ml), 1 ml/l; Antifoam solution, 1.5 ml/l
Prebiotic MIX	Arabinoglactan, 2 g/l; Pectin from apple, 2 g/l; Starch from potato, 5 g/l; Xylan from corncob, 2 g/l; Inulin from dahlia tubers, 1g/l; Beta-glucan, 0.5 g/l; Glucose, 2 g/l;<br>Mucin from porcine stomach, 4 g/l

Table S2. Pairwise comparison of beta diversity between treatments in CM and LS subjects at T16 and T24

	Groups	measure	F	R2	p.value	p.adjusted	Significance
**CM_T16**	basal vs. MIX	bray	2.24	0.14	0.007	0.014	*
	basal vs. MIX+S7	bray	3.39	0.19	0.001	0.003	**
	basal vs. basal+S7	bray	0.76	0.05	0.535	0.559	ns
	MIX vs. MIX+S7	bray	0.77	0.05	0.559	0.559	ns
	MIX vs. basal+S7	bray	1.90	0.12	0.04	0.06	ns
	MIX+S7 vs. basal+S7	bray	3.25	0.19	0.001	0.003	**
**CM_T24**	basal vs. MIX	bray	2.05	0.13	0.032	0.048	*
	basal vs. MIX+S7	bray	2.80	0.17	0.004	0.012	*
	basal vs. basal+S7	bray	1.68	0.11	0.102	0.1224	ns
	MIX vs. MIX+S7	bray	0.81	0.05	0.526	0.526	ns
	MIX vs. basal+S7	bray	3.06	0.18	0.007	0.014	*
	MIX+S7 vs. basal+S7	bray	4.42	0.24	0.001	0.006	**
**LS_T16**	basal vs. MIX	bray	2.55	0.15	0.051	0.27	ns
	basal vs. MIX+S7	bray	1.91	0.12	0.102	0.27	ns
	basal vs. basal+S7	bray	1.35	0.09	0.249	0.3735	ns
	MIX vs. MIX+S7	bray	0.96	0.06	0.47	0.47	ns
	MIX vs. basal+S7	bray	2.02	0.13	0.135	0.27	ns
	MIX+S7 vs. basal+S7	bray	0.67	0.05	0.411	0.47	ns
**LS_T24**	basal vs. MIX	bray	3.29	0.19	0.01	0.06	ns
	basal vs. MIX+S7	bray	2.07	0.13	0.114	0.228	ns
	basal vs. basal+S7	bray	0.33	0.02	0.709	0.709	ns
	MIX vs. MIX+S7	bray	0.95	0.06	0.432	0.5184	ns
	MIX vs. basal+S7	bray	2.63	0.16	0.049	0.147	ns
	MIX+S7 vs. basal+S7	bray	1.45	0.09	0.225	0.3375	ns

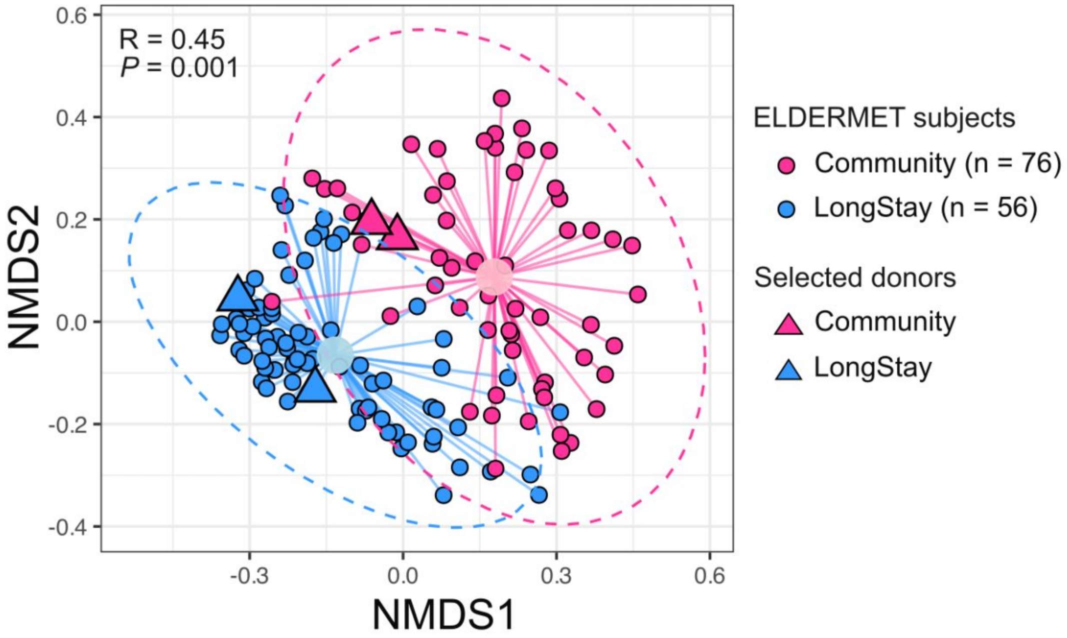

Figure S1. Nonmetric multidimensional scaling (NMDS) ordination of fecal microbiota in community-dwelling (CM) and long-stay-dwelling (LS) older subjects. The Bray-Curtis index were performed at the ASV level to generate NMDS to visualize gut microbiota similarities between CM and LS samples. The microbiota of selected donors (triangles) were compared to that of a subset of the ELDERMET cohort (circles: CM, n = 79; LS, n = 59). Each data point represents the microbiota of one sample. The ellipses represent the standard deviation of data points belonging to each group, with the center points of the ellipses calculated using the mean of the coordinates per group.

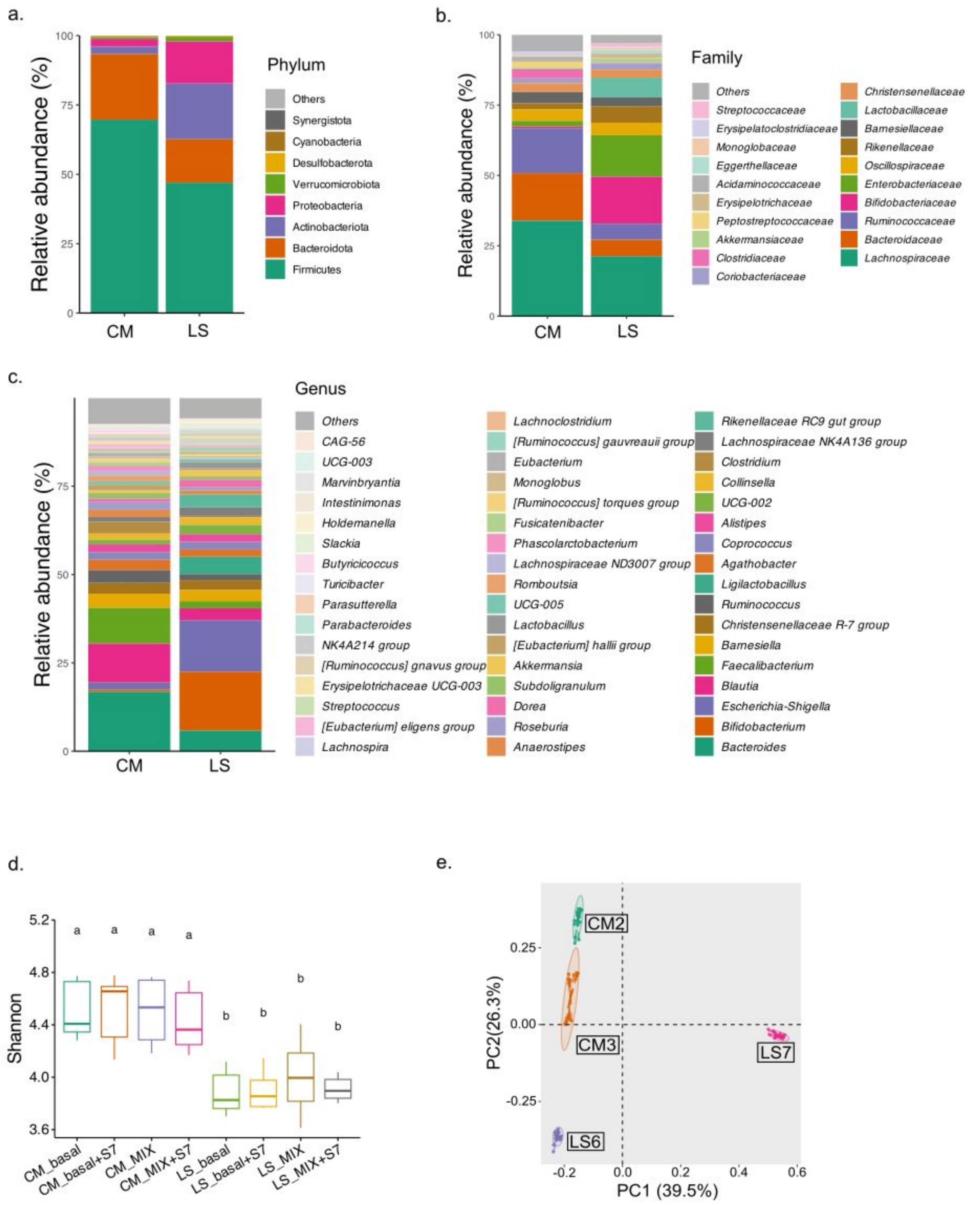

Figure S2. Microbiota compositions of donors at baseline. Comparison of gut microbiota composition (mean relative abundance) between CM and LS donors at the phylum (**a**), family (**b**), and genus (**c**) levels. **d.** Boxplot of alpha diversity as measured by Shannon index (n = 8 per group). Different letters (a, b) indicate statistically significant ($P < 0.05$) differences between groups. **e.** Beta diversity based on Bray-Curtis distances. CM, community-dwelling; LS, long-stay-dwelling.

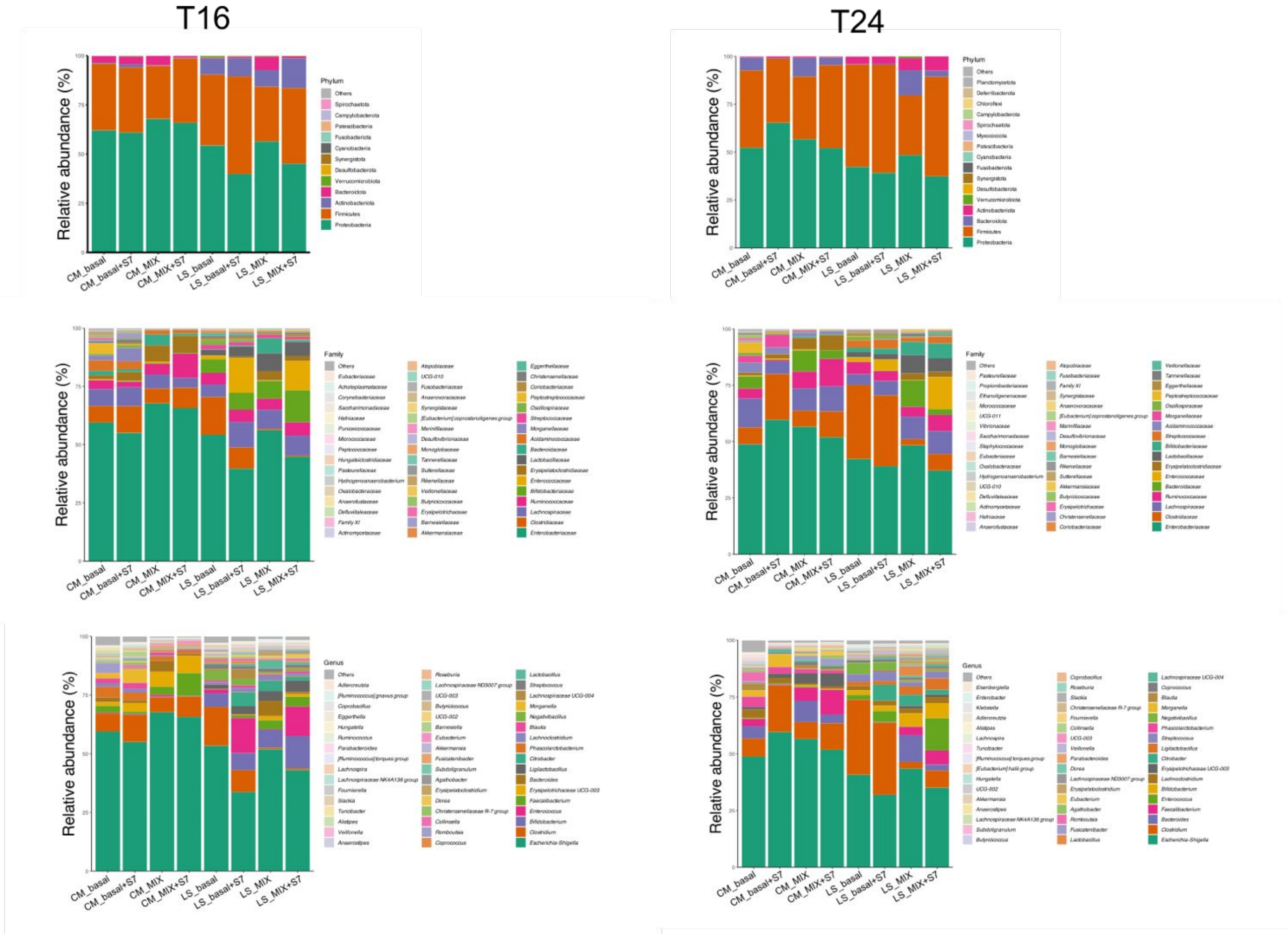

Figure S3. Microbial compositions after 16h and 24h fermentation. Mean relative abundances of microbiota from each treatment group (n = 8) at phylum, family, and genus level.
